# Noise Genetics: Inferring Protein Function by Correlating Phenotype with Protein Levels and Localization in Individual Human Cells

**DOI:** 10.1371/journal.pgen.1004176

**Published:** 2014-03-06

**Authors:** Shlomit Farkash-Amar, Anat Zimmer, Eran Eden, Ariel Cohen, Naama Geva-Zatorsky, Lydia Cohen, Ron Milo, Alex Sigal, Tamar Danon, Uri Alon

**Affiliations:** 1 Department of Molecular Cell Biology, Weizmann Institute of Science, Rehovot, Israel; 2 Department of Plant Sciences, Weizmann Institute of Science, Rehovot, Israel; Washington University School of Medicine, United States of America

## Abstract

To understand gene function, genetic analysis uses large perturbations such as gene deletion, knockdown or over-expression. Large perturbations have drawbacks: they move the cell far from its normal working point, and can thus be masked by off-target effects or compensation by other genes. Here, we offer a complementary approach, called noise genetics. We use natural cell-cell variations in protein level and localization, and correlate them to the natural variations of the phenotype of the same cells. Observing these variations is made possible by recent advances in dynamic proteomics that allow measuring proteins over time in individual living cells. Using motility of human cancer cells as a model system, and time-lapse microscopy on 566 fluorescently tagged proteins, we found 74 candidate motility genes whose level or localization strongly correlate with motility in individual cells. We recovered 30 known motility genes, and validated several novel ones by mild knockdown experiments. Noise genetics can complement standard genetics for a variety of phenotypes.

## Author Summary

Inferring the function of proteins and the role they play in cellular processes is essential for our understanding of cell biology, genetics and biology in general. Standard genetic approaches use large perturbations to cells such as gene knockout, knockdown or over expression of genes. Such methods are powerful, but have the drawback of taking the cell far from its normal working point. Here, we provide a new and much milder approach, which uses the natural cell-cell variation in protein level and expression pattern as a source of mild perturbation. We monitor individual live cancer cells under the microscope and correlate their protein levels and localization with phenotype in the same cells. We use the motility of human cancer cells as a model system that is highly important for understanding metastasis in cancer. We find that our approach uncovers most of the known motility proteins, as well as new ones which we validate using knockdown experiments. Our novel approach is widely applicable to any phenotype that can be visualized in individual cells, and for any organism for which one can measure proteins in individual cells.

## Introduction

To understand which proteins contribute to a biological phenomenon, current approaches use perturbations such as gene knockdown, over-expression or knockout. These approaches have provided the basis for much of what we know about cell biology. However, such perturbations also have drawbacks. Perturbations currently used are usually large - a protein expression is either markedly reduced or increased, and the measurement is therefore far from the cells normal working condition. This can lead to artificial off-target effects or to masking of the perturbation by changes in the cell that compensate for the loss of a protein. It is thus possible that some of the information about protein function has remained hidden due to these features of current methods.

To offer a complementary way to understand protein function, we present an approach called noise genetics. Noise genetics uses the natural cell-cell variation in protein levels and localization [1]–[8] as a source of mild perturbations to reveal protein function. Since natural fluctuations are mild, the risk of compensation is reduced. The idea is to correlate the protein levels and localization in individual cells to the phenotype in the same cells. Notably, cell-cell variation in protein level changes slowly over time: cells keep their individual levels for about a cell generation [7]. Thus, the noise we use is a type of cell individuality (Figure S1). Cells have individual character in many of their phenotypes as well, that also last for about a cell generation [9], [10].

Previous studies used noise for understanding regulatory interactions between a few proteins in bacteria [1], [11], whereas here we screen hundreds of proteins. As a model system, we use the motility phenotype of human cancer cells. Motility of cancer cells is of general interest both as a well-studied biological phenotype [12], and as a feature of normal physiology and cancer metastasis [13]. Wide scale genetic screens, including siRNA knockdowns, have revealed numerous genes involved in motility [14]–[16]. Moreover, natural phenotypic variability and fluorescent microscopy were used to study the shape of motile cells [10] and the cytoskeleton dynamics [17].We use a library of human cancer cell clones each with a different protein fluorescently tagged at its endogenous chromosomal locus [18], [19] to follow the natural variability of proteins, and the natural variability of motility in the same cell. Proteins whose level or localization correlate with motility are identified as candidate motility proteins.

We find that about 15% of the 566 highly expressed proteins that we tested exhibit a significantly high correlation between their protein features and motility in individual cells. This correlation can suggest that the protein has a role in cell motility. About half of these candidate proteins were previously known to be involved in cell motility. We validated a sample of these candidates using mild siRNA knockdown.

## Results

### Level and localization of 566 unique proteins were analyzed in time-lapse movies

**Figure 1:**
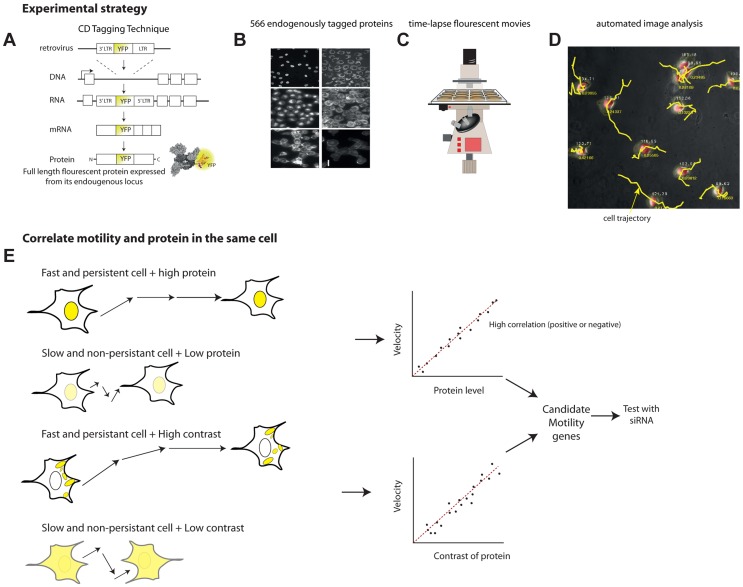
Overview of the noise genetics approach. (a) We used a library of endogenously tagged proteins in human H1299 cells. Briefly, a retrovirus introduced YFP as an artificial exon into the introns of genes. Fluorescent clones were selected and sequenced. The clones express full length protein under endogenous control, with an internal YFP tag. (b) We selected 566 unique proteins with high quality movies and correct localization, and performed, or used existing, 24 h time-lapse movies(c) under controlled conditions. (d) Automated image analysis enabled by a mCherry tag in the parental clone enabled automatic tracking of protein level and localization as well as motility of each individual cell over time. (e) To find candidate motility genes, we sought proteins with high absolute correlation between protein level or localization (contrast, texture) and motility parameters (velocity, persistence). We tested a sample of the candidate motility genes by siRNA knockdown.

To study natural variability between individual cells, we used the LARC library of human clones with tagged proteins [18]–[20]. The library is made of clones of a parental human lung cancer cell line, H1299. In each clone, a full-length protein is fluorescently tagged with YFP as an internal exon (Figure 1A). The protein is tagged at its endogenous chromosomal locus, preserving the natural promoter and regulatory sequences (Figure 1B). Previous studies suggest that most (70–80%) of the tagged proteins preserve their wild-type dynamics and localization [18], [20].

The parental clone also expresses proteins tagged with red florescence using mCherry. This red fluorescence is used for image analysis of time lapse movies (Figure 1C), allowing automated segmentation and tracking of the nucleus and cytoplasm in all clones (Figure 1D). The tagged proteins are XRCC5 and DAP1, both not known to be involved in motility.

A previous study employed this library to follow 1260 clones with different tagged proteins as they responded to an anti-cancer drug using time-lapse movies [18]. Here, we re-analyzed these movies, that also included the 24 h period before drug addition, together with movies from a recent study on protein half-lives using the same library and microscopy system (Eden *et al*, 2011) and chose 704 unique proteins with high quality movies for further analysis (movies chosen had 4 fields of view totaling at least 20 cells at each time-point). Of these, we chose only known proteins (as opposed to ESTs) with subcellular localization matching the literature. This results in a final set of 566 different proteins. These proteins have diverse cellular localizations and functions (Supplementary File S1).

### Correlation between protein and motility features was tested for each cell

We tracked the protein level and localization in each cell, and also the motility of the same cells. Protein level is given by the summed YFP fluorescence of all pixels in the cell. Most proteins in our dataset did not show large translocation events between cell compartments such as nucleus and cytoplasm. To parameterize protein localization, we therefore characterized the spatial distribution inside the cell, using two well-known measures from image analysis: contrast (the existence of sharp changes in intensity) and texture (also called texture correlation, the linear dependency of grey levels on those of neighboring pixels) [21]–[23].

**Figure 2:**
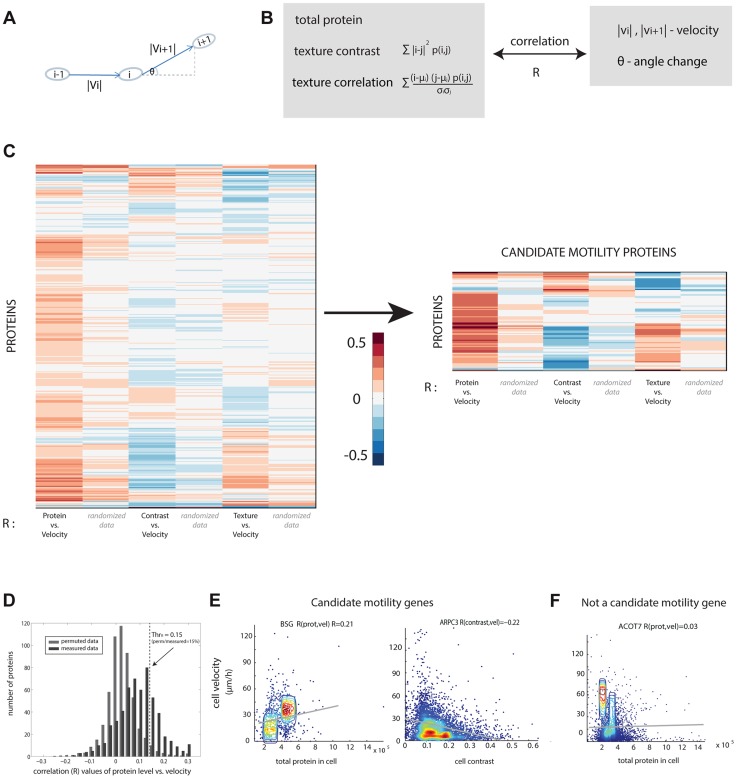
Candidate motility genes were found with high correlation between protein and motility features in individual cells. (a) Cell velocity and angle change (persistence) for a cell in three consecutive frames i−1,i and i+1. (b) Three protein features were compared to two motility features. Contrast and texture sum over pixels in a cell and employs the occurrence matrix p (i, j) equal to the probability that pixels of intensity i and j are adjacent. Texture uses the mean and standard deviation (std) of pixel intensity levels mu and sigma. (c) Pearson correlation coefficients R between protein and motility features show a group of proteins with high absolute correlation. Blue/red denotes low/high R values. (d) Randomized data (gray) showing lower correlations than real data (black) is used to establish a threshold correlation (dashed line) for detecting motility candidate genes with low false discovery rates. (e) Candidate motility genes show positive or negative correlation to protein level or localization measures. Data is shown in density plot along with the regression line of the linear fit. (f) An example for a non-motility candidate that show low absolute correlation.

Cell motility was quantified in terms of speed and angle change of the motion (Figure 2A). Cell velocity was measured as the change in cell center of mass between frames (20 min). The angle change of cell movement in frame i was calculated based on the deviation of the cell in frame *i+1* from its movement between frame *i-1* and frame *i*. Persistent motion results in low values of angle change (Figure 2B). We found that angle change was negatively correlated with cell velocity (Figure S2).

If a protein is involved in motility, we expect a significant positive or negative correlation between at least one of the protein parameters and one of the motility parameters. For example, if cells that express protein X at high levels move faster, whereas cells that express it at lower levels move slower, we predict that protein X is a candidate motility gene (Figure 1E). A similar conclusion is reached if contrast or texture correlate with motility. For example, if cells that show a homogenous spatial distribution of protein Y move slower than cells that express protein Y in more punctuate manner (high contrast of fluorescence across the cell), one may predict that Y is a candidate motility gene. The contrast and texture differences we observe between cells are subtle, not gross changes such as transitions between organelles. Images of cells are provided in S3–S5.

We compared the three protein properties (protein level, contrast and texture) to the two motility parameters (speed, angle change) in each cell and each time-point using Spearman and Pearson correlations (Figure 2C, Figure S6). The observed correlation values are centered at around zero and range from −0.4 to 0.4.

In order to test the significance of the calculated correlation values, we compared them to correlations in randomized data. To make a stringent comparison, we note that data from different time-points of the same cell are not independent. Furthermore, cells from the same field of view are potentially more dependent than cells in other fields of view, due to possible systematic effects in the experiment. We thus constructed the randomized dataset by associating the motility parameters for cell i at all time points with the protein properties from a different cell j at the same time points in the same field of view, with i and j randomly chosen. This provides a randomized control of the same size as the original data.

The permuted datasets showed correlations between protein and motility parameters mainly (90%) in the range −0.1 to 0.1 (gray bars in Figure 2D). Comparing the randomized distribution to the measured data (black bars in Figure 2D) shows proteins with correlations higher or lower than expected by chance. In this study, we define a candidate motility gene if its absolute correlation coefficient |R| exceeds 0.15 in both Pearson and Spearman correlations. Based on the comparison to randomized data, the rate of false positives is expected to be 15% for comparisons of protein level to cell velocity (Figure S7), 23% for contrast versus velocity, 30% for texture versus velocity. To test this, we conducted a new set of time-lapse microscopy experiments on a random sample of 19 candidate motility proteins, and found that 16/19 showed the same above-threshold correlations as in the original movie dataset, consistent with a false-detection rate of about 15% (see Supplementary File S3).

Similar false discovery rates were obtained for the comparison between angle change and the protein properties (Figure S7). Several examples of proteins with positive and negative high correlations are shown (Figure 2E) along with examples of proteins that did not show a significant correlation between the protein level and the motility in individual cells (Figure 2F).

### Many of the motility candidate genes are known to play a role in motility

We found 74 candidate motility genes (Supplementary File S1, S2). Of these, 31 (41%) correlate by protein level. The rest of the proteins correlate with motility by contrast (25%) or texture (23%), and 15% of the candidate proteins correlate by more than one measure.

**Figure 3:**
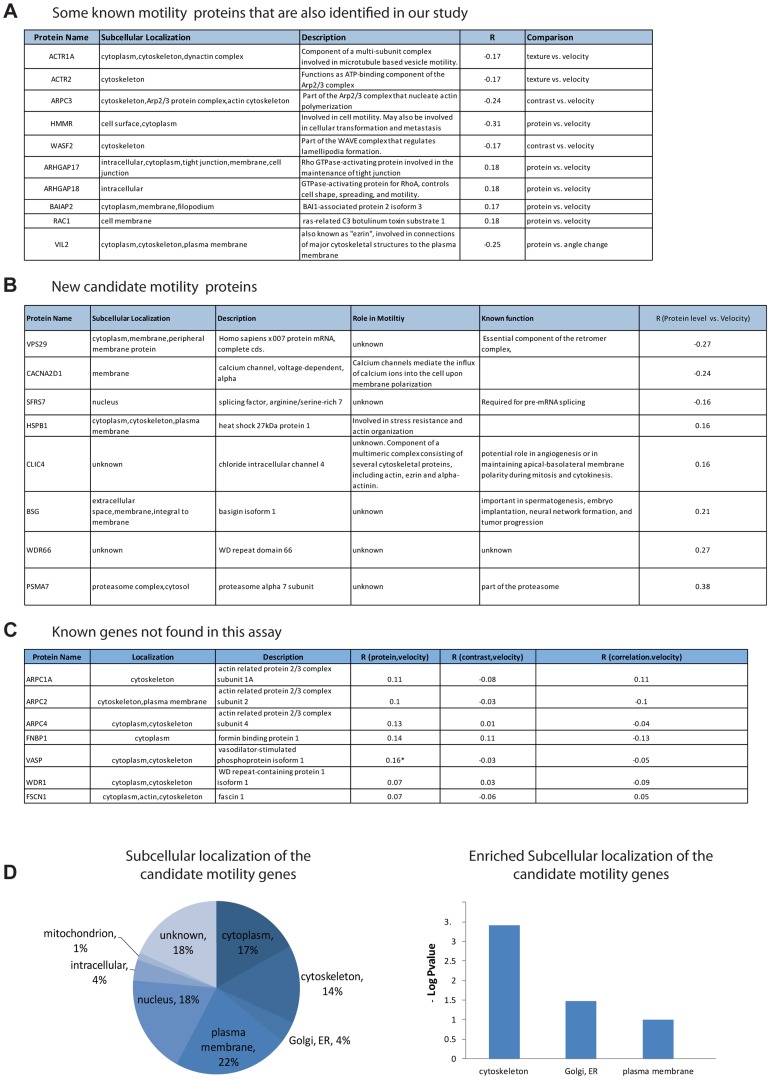
Candidate motility genes recover many of the known motility proteins in the screen. (a) Examples of known motility genes found in the present assay, with their correlation coefficient. (b) New candidate motility genes found in the assay. (c) Known motility genes included in the screen but not found in the assay. Note that three of the genes have marginal R values. (d) Sub-cellular localization of candidate motility genes is enriched for cytoskeleton, Golgi/ER and plasma membrane, similar to all known motility genes (gene-cards).

The candidate genes are highly enriched in genes previously known to play a role in motility: 41% (30/74) were previously characterized as motility genes (hypergeometric p = 0.0009) according to the Genecards database (see Methods). Among the candidate genes are actin regulators in the ARP complex (ARPC3 [24]), two actin related proteins (ACTR2 and ACTR1A), RAC1 [25] that is essential for cell migration and WASF2 [26] that is part of the WAVE complex that regulates lamellipodia formation [12]. (Other examples are described in Figure 3A). Some of the candidate genes have no known role in motility (Figure 3B).

The subcellular localization of the candidate genes is enriched in the cytoskeleton (hypergeometric p-value = 0.0008), the plasma membrane (p = 0.02) as well as the ER/golgi (p = 0.06) (Figure 3D).

In an attempt to estimate the false-negative rate of this assay, we considered the 13 genes out of the 566 in this study that were listed as motility genes in a recent review of motility (Ridley, 2011). Among these 13 relatively well-characterized genes, 7 genes were not identified as candidate motility genes in this study (Figure 3C). This suggests a false-negative rate on the order of 50%. However, some of these false negative genes showed a relatively high correlation in one of the examined comparisons and would be scored positive in a less stringent threshold choice. A more extensive false-negative test compared the present assay to all 110 genes in our set of 566 that are listed as motility related in the Genecards database. Of these, only 29 are found in our assay. Our analysis is not expected to find all motility genes due to several limitations that will be addressed below.

### Mild knockdown experiments were used to validate some of the candidate genes

**Figure 4:**
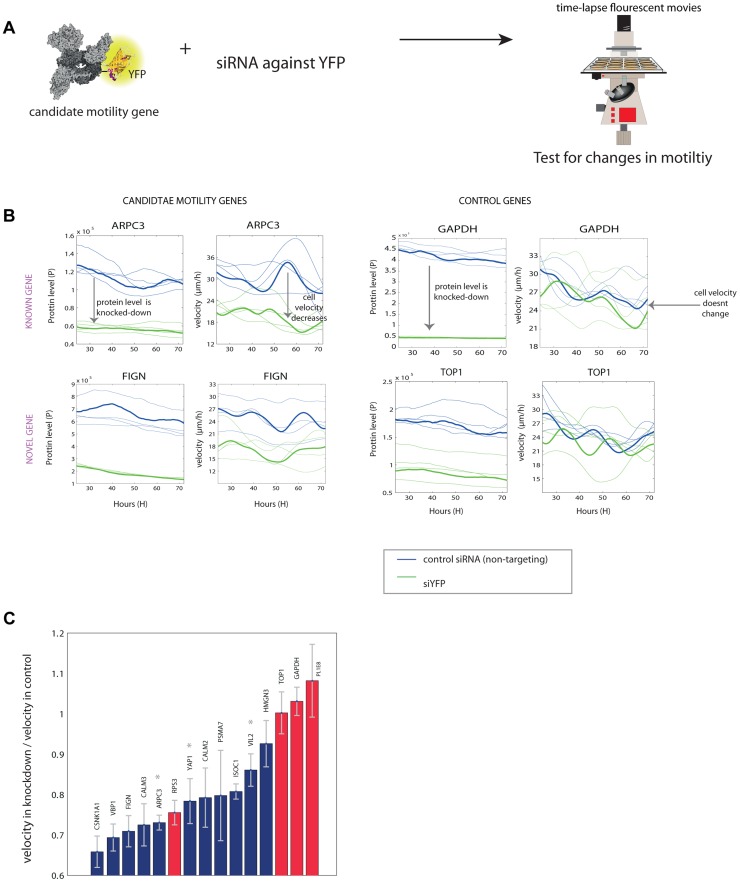
Novel motility genes are validated by mild knockdown experiments. (a) We used anti-YFP siRNA to produce knockdown of the tagged gene in each clone, and tested protein level and motility in 48 time-lapse movies. (b) Examples of known and novel candidate motility genes after knockdown, as well as control genes, that are not known to be involved in the motility process. On the left, the effect of the knockdown on the expression of the protein is shown. In all knockdown experiments, protein fluorescence level was reduced at least by half. On the right, the effect of the knockdown of the gene on cell speed is shown. In the examples shown, candidate motility genes showed a reduction in velocity after knockdown compared to mock treatment, whereas control genes showed no significant reduction in motility. (c) Velocity reduction in knockdown experiments compared to mock treatment shows that 10/11 candidate genes showed a motility defect upon knockdown (blue bars), in contrast to control genes not known to be involved in motility, for which 3 out of 4 showed no significant defect (red bars). Stars denote known motility genes also found in our assay. Error bars stands for standard deviations (SD).

In order to test the involvement of the candidate genes in the motility process, we used siRNA directed against the YFP tag to lower the expression level of the tagged proteins. Since we introduced YFP as an exon to all clones, the anti-YFP siRNA can be used to knockdown expression in any clone from the library. The knockdown is mild (at most half-knockdown) because only one allele is tagged with YFP. Thus, a 50% reduction in YFP corresponds to a 25% reduction in total protein due to the expression from the untagged allele. We chose 11 candidate genes that had positive correlation between protein level and motility or a significant correlation between a protein feature and motility. Of these candidate genes, 4 are previously known motility genes and 7 are novel, randomly chosen from the candidate list. We also tested 4 control genes that did not correlate with motility. We took time-lapse movies for 48 hours starting 24 hours after the siRNA infection (Figure 4A).

We used the YFP fluorescence to observe the extent of knockdown (Figure 4B). The mCherry labeling of the cells was not influenced by the siRNA (Figure S8). 10 out of the 11 candidate motility gene clones showed significantly lowered velocity (reduction of 15–35%), whereas 3 of the control genes (GAPDH and TOP1 and one gene of unknown function) showed no measurable reduction in velocity (Figure 4C).

One control gene – the ribosomal gene RPS3, not known to affect motility, showed a motility defect upon mild knockdown. It was not picked up as a candidate motility gene in the noise genetics assay based on cell-cell variations. This may point to a difference between knockdown that affects only a single gene in a module such a ribosome, and noise genetics, where fluctuations in all genes in a module (e.g. all ribosomal genes) are expected to be correlated [7], [8].

In total, candidate motility genes, both previously known and novel were validated by mild knockdown at a level of about 90%. We further tested proteins with a negative correlation between their protein level and motility and the results are summarized in the supplementary information (see Supplementary File S4).

## Discussion

In this study, we presented ‘noise genetics’ - an approach to assign function to proteins that uses the natural noise in protein level and localization and correlates it to the variation in phenotype of the same individual cells. We demonstrated this using the motility phenotype of cancer cells. Noise genetics recovers 30 of the known motility genes in our clone library and also 43 novel motility candidates, of which 10 were validated by siRNA knockdown (Figure 4).

Noise genetics can complement standard genetic perturbations. Among its advantages are the non-invasive and mild nature of the natural variations used, which keep the cell near its normal working point. Proteins whose knockdown is lethal are hard to evaluate using standard genetics, but can potentially be picked up by the assay. Similarly, proteins whose knockdown effects are masked by compensation from other proteins in the same module may be picked up by noise genetics, because one expects the entire module to show correlated noise [7], [8]. Noise genetics compares individual cells from the same field of view, and therefore contains a type of internal control for systematic errors. In standard perturbation assays, one needs to compare perturbed cell populations to a separate experiment with unperturbed cell populations in order to control for experimental systematic errors.

Among the limitations of noise genetics as implemented here are the need for a fluorescent cell library or other means of observing cell-cell variation in both protein and phenotype. Such libraries exist, for example, in *S. cerevisae* [27], *E. coli* [28], [29], *C. elegans* [30] and Zebrafish [31]. If the phenotype of interest can be observed in fixed cells, individual cell imaging of proteins [32] or mRNA [33]–[35] might be used for noise genetics. Such an approach has been used with pre-selected genes, for example, to explore the effect of protein variability on stem-cell differentiation [36], sporulation timing in Bacillus subtilis [37] and meiosis timing in Saccharomyces cerevisiae [38]. We currently tested only linear correlation; more elaborate time-series analysis methods or non-linear correlation analysis may be able to improve the resolution of this approach [39].

The cells in this study are diploid (or multiploid), but only one copy of each gene was labeled with YFP. Therefore, the fluorescence measurement does not necessarily reflect the total protein level or distribution. Previous work with the present cell system showed that there is high correlation in the expression of two alleles of the same ribosomal gene [7]. Future work is needed to test the present approach with all alleles tagged.

Noise genetics can miss proteins whose effect on phenotype is small in the working point of the cell. Such proteins can be picked up by standard genetics which makes large perturbations. Similarly, if natural variations in proteins or phenotypes are very small, noise genetics may not be applicable. Importantly, noise genetics on its own can only detect correlations, and additional experiments such as the mild knockdown performed here, are needed to gain evidence for causality.

Noise genetics uses natural cell-cell variation in proteins to discover links with phenotypes. Additional phenotypes that can be readily studied include cell size and shape, and any other phenotype measurable by time-lapse microscopy.

## Methods

### Time-lapse microscopy movies

Movies from previous studies [18], [20] were used for this analysis, as well as new movies on 19 clones. In the previous studies, 4 movies (fields of view) were taken for each of the 1,000 clones totaling about 4,000 movies. In each movie, 10–20 cells were tracked over 24 hours at least, every 20 minutes. Some of these clones were filmed more than once and some clones represent the same protein. For the present analysis, we combined all data for the same protein from all relevant movies. Each time point included transmitted light image (phase contrast) and two fluorescent channels (red and yellow). Of the original movie sets, we chose 566 unique protein clones as described in the text. The 566 known proteins that were used in our analysis tend to have high expression levels (so that they are picked up in the LARC library construction which used FACS to select for fluorescent clones).

### Image analysis of time-lapse movies

We used the image analysis software described in [18] with minor modifications. The main steps in this software include background correction (flat field and background subtraction), segmentation, cell tracking, and automated identification of cell phenotypes (mitosis and cell death). Cell and nuclei segmentation was based on the red fluorescent images of the two red tagged proteins found in all clones, localized to the cytoplasm (DAP1) and nucleus (XRCC5), with intensity which is very uniform across cells and clones. Segmentation used global image threshold and seeded watershed segmentation. The cell-tracking procedure maps each cell to the appropriate cells in the preceding and following frames as described [40]. Texture parameters (contrast and correlation) of the proteins were measured for each cell in each time point based on the YFP image of the tagged protein as described below.

In our previous studies with the same movies, we also analyzed the protein concentration by taking the average or median fluorescent intensity inside the cell, as opposed to the total intensity. We find this measure to be more sensitive to image outlier pixels - even when using the median pixel intensity [23]; we therefore use total fluorescent intensity in the present study.

### Texture and contrast calculation

To calculate texture and contrast, we first evaluated a gray-level (fluorescence intensity) co-occurrence matrix (GLCM) from each fluorescent image of the cells [41]. Each element (i, j) in GCLM specifies the number of times that the pixel with gray-level i occurred horizontally adjacent to a pixel with gray-level j.

From the matrix one can compute the various texture features. For example, contrast is 
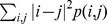
 giving a value of 0 for a constant intensity image and high values when adjacent pixels have different intensity. Our second measure, ‘texture’ or ‘correlation texture’ is 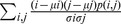. The correlation texture measures the linear dependency of grey levels on those of neighboring pixels. The two measures are weakly anti-correlated (Fig. 2C). We verified that the texture and contrast values that were calculated after removing the background and after rescaling to 64 gray levels have a low sensitivity to rotations (Figure S5).

### Correlation analysis

For each protein, we collected all cells in all fields of view at all time frames. For each cell, 3 protein parameters (total protein, contrast and texture) and 2 motion parameters (velocity and angle change) (See Figure 2A) were calculated. Then, 6 correlation values (between all pairs) were calculated using Pearson and Spearman correlation. No binning was used to compute these correlations. The correlation values are summarized in Supplementary File S1. We tested other texture features as well (homogeneity and energy), but these did do not add known genes to the list of candidates (data not shown). Next, we collected only cells that were tracked from the beginning of the movie until its end and repeated the same calculation.

In order to establish a threshold that minimizes the false positive rate, we generated permuted datasets. In each permuted dataset, for each of the protein parameter and for each of the motion parameter, the protein values from one cell time trace were correlated to the motion values in another cell time trace in the same field of view. We repeated this permutation 10 times for each of the 6 comparisons (and both for Pearson and Spearman calculations) and generated 6 correlation distributions, for all proteins. These correlation values are summarized in the Supplementary File S3. We chose to use both Spearman and Pearson correlations since using only Spearman correlation results in marginally higher false positives (Data not shown). A correlation value of R = 0.15 (or similar) was chosen in order to minimized false positives, while maximizing potential true candidates (See Figure S7). We further tested for all the proteins with significant correlation values in which the correlation still holds even when calculating it only in one field of view (FOV) for most of its FOVs. The final candidate list includes only proteins that passed this test.

The shape of the cell is known to affect its motility [10]; in order to estimate this effect in our dataset, we calculated the correlations between the aspect ratio (major axis/minor axis) of each cell and the cell velocity and found no significant correlation in the examined clones (Figure S9).

### Validation analysis

We conducted new experiments by performing time-lapse movies for 19 of the candidate clones. Of the 19, 13 proteins were chosen randomly out of the list of novel candidate proteins, and the other were candidates that were also known motility genes. 16 out of the 19 showed a correlation that is similar to the previous calculated correlation. (Results are shown in Supplementary File S3).

### Function analysis

Several databases were used to annotate whether genes are known to be involved in motility (Supplementary File S2). We used GeneCards (http://www.genecards.org/) to download all genes with the keywords “motility” or “migration”. We further considered genes that were identified as part of the “adhesome” (http://www.adhesome.org/). Finally, well-studied characterized genes listed in the Review by [12] were also considered as motility genes.

### Subcellular localization assignment and enrichment test

We used subcellular localization according to GeneCards (http://www.genecards.org/) and other databases as provided in the LARC database (http://www.weizmann.ac.il/mcb/UriAlon/DynamProt/). All clones used here have localization in the experiment that agrees with the previously known localization. When more than one localization was assigned to a protein, the first localization in the list was used for the category assignment. The subcellular information and categories are list in the “Candidate_genes.xls” file. Hypergeometric p-value [42] was used to calculate the enrichment of specific subcellular localization categories in the candidate genes group over all the 566 genes used in this analysis.

### Mild knockdown experiments

To knockdown the expression of the tagged protein in clones from our library, we used siRNA against GFP (QIAGEN, 1022064) transfection using lipofectamin (Invitrogen) as described in their protocol. As a control siRNA, we used the non-targeting siRNA (Dharmacon, D-001810-10-05). No significant difference between siRNA used from QIAGEN or Dharmacon was detected in our system (Figure S10).

Briefly, 2×10^4^ cells were grown on 12-well glass bottom MatTek plates. The next day, siRNA transfection was performed. We used 24 pmol of si-RNA for each well and 0.8 ul lipofectamin and incubated it for 6 hours, then we replaced with fresh media and let cells grow overnight. The next day, we took a time-lapse movie of the plate for 48 hours. We took fields of view from each well. We used the same exposure time for the well with the non-targeting siRNA (where no decrease in expression is expected) and the well with the si-GFP (which showed a decrease in the YFP fluorescent and not in the mCherry fluorescent).

## Supporting Information

Figure S1 Cell individuality. (A) Protein level dynamics in individual cells (NOL7 and ARPC3 clones) are shown. Note that if a cell has higher than average or lower than average level of a protein, it remains so for about a cell generation or more. (B) Contrast level of individual cells are shown in the ARPC3 clone. Again, though contrast level varies along time, cells have correlation times of about a cell cycle. (C) Autocorrelation function for 2 single cell trajectories of the NOL7 protein to illustrate cell individuality.
(PDF)

Figure S2 Correlation between velocity and angle change. The velocity and angle change is shown for thousands of cells. Note that a general anti-correlation is evident.
(PDF)

Figure S3 Images of individual cells. Images of individual cells from different clones are shown along with their contrast value (on the left) and their velocity (µM/20 minutes). The white point represents the location of the cell in the previous frame, the light blue point represents the cell location in the current frame and the blue point represents the location in the next frame.
(PDF)

Figure S4 Different individual cells of the ARPC3 clone in one field of view. Four different cells from the ARPC3 clone are shown along with information about their contrast values and their different trajectory along 13 consecutive frames (frames taken every 20 minutes).
(PDF)

Figure S5 Texture features calculations of images. (A) Cell images from the ARPC3 clone are shown along with their contrast and texture correlation values, calculated without rotation and after averaging the contrast and texture correlation values (after 4 rotations). The contrast is plotted against the velocity for the ARPC3 clone when calculating the contrast without rotation (C) and after averaging the contrast values after 4 rotations (D) A high correlation (R = 0.99) is evident between the contrast values without rotation and the average contrast values after rotation. (E) A high correlation (R = 0.97) is evident between the texture correlation values without rotation and the average texture correlation values after rotation. Similar results were obtained for the different clones.
(PDF)

Figure S6 Correlation coefficients R between protein and motility features. Similar to Figure C2, a correlation coefficients matrix between the 3 protein parameters and the two motility features is shown. On the right, a group of proteins with high absolute correlation is shown. Blue/red denotes low/high R values.
(PDF)

Figure S7 Comparisons between the real and permuted correlation values. The correlation values calculated from the real dataset are in dark grey, while the correlation values calculated from 10 permuted datasets are in light grey. This analysis helped us to choose a threshold that would minimize the number of hits in the permuted dataset compared to the number of hits in the real dataset. The chosen threshold for each comparison is written on the right of the plot.
(PDF)

Figure S8 Knockdown experiments specifically decrease the expression of the YFP tagged protein and not the mCherry tagged protein. A typical field of view of the ARPC3 clone is shown after si-GFP experiment and after the control experiment (with non-targeting si). The parental clone has 2 proteins tagged with mCherry to help with the segmentation of the nucleus and the cytoplasm. No decrease in mCherry expression is evident. However, the ARPC3 is tagged with YFP and a significant reduction is shown in the YFP expression. On the bottom, a quantification of 4 different FOVs of the ARPC3 clone demonstrates similar results. Similar results were obtained for all the examined clones.
(PDF)

Figure S9 Aspect ratio does not correlate with velocity in our system. The aspect ratio of single cells, a measure that describes cell shape, was plotted against the velocity of cells in the same clones as in figure 2F, E. No significant correlation is evident for any of these clones.
(PDF)

Figure S10 Control knockdown experiments. GAPDH clone was used as a negative control since it is not a candidate motility gene. siRNA against GAPDH (Dharmacon) was used, as well as siRNA against GFP (QIAGEN) that should target any gene in our library that is tagged with YFP and also non-targeting siRNA (Dharmacon) that is not targeted against any specific gene and is widely used as a negative control. As expected, no significant change in the velocity was observed between these 3 conditions. Therefore, in all our following experiment, we used the siGFP that is expected to lower the expression of the target gene and the non-targeting siRNA that serves as a negative control for the siRNA experiment.
(PDF)

Figure S11 Negative correlation between protein and motility. Knockdown experiments were carried out for 4 genes that showed a negative correlation between protein level and motility. Analysis was done as described in Figure 4C. Velocity reduction in knockdown experiments compared to mock treatment shows that 4/4 candidate genes showed a motility defect upon knockdown (blue bars), in contrast to control genes not known to be involved in motility, for which 3 out of 4 showed no significant defect (red bars). Stars denote known motility genes also found in our assay. Error bars stands for standard deviations (SD).
(PDF)

File S1 Correlation values. Pearson and Spearman correlations values are shown for all the genes in our dataset for all six comparisons.
(XLSX)

File S2 Candidate motility genes. 74 candidate motility genes are shown along with their correlation values (both Spearman and Pearson), correlation values after binning, gene description, subcellular localization, etc.
(XLSX)

File S3 Tacked cells and permuted values. Pearson and Spearman correlations values are shown for all the genes in our dataset only for cells that were tracked along all frames for all six comparisons. Correlation values after the permutation are also shown. The results of the validation analysis of the 19 genes are shown as well.
(XLSX)

File S4 Negative correlation between protein and motility. A short discussion on genes in our analysis that presented a negative correlation with motility.
(DOCX)
