# Supplementary material for: Noise Genetics: Inferring Protein Function by Correlating Phenotype with Protein Levels and Localization in Individual Human Cells
Source: PLoS Genet. 2014 Mar 6;10(3):e1004176. doi: 10.1371/journal.pgen.1004176 (PMC3945223; doi:10.1371/journal.pgen.1004176)
Supplement: Figure S1 — Cell individuality. (A) Protein level dynamics in individual cells (NOL7 and ARPC3 clones) are shown. Note that if a cell has higher than average or lower than average level of a protein, it remains so for about a cell generation or more. (B) Contrast level of individual cells are shown in the ARPC3 clone. Again, though contrast level varies along time, cells have correlation times of about a cell cycle. (C) Autocorrelation function for 2 single cell trajectories of the NOL7 protein to illustrate cell individuality. (PDF) [file pgen.1004176.s001.pdf]

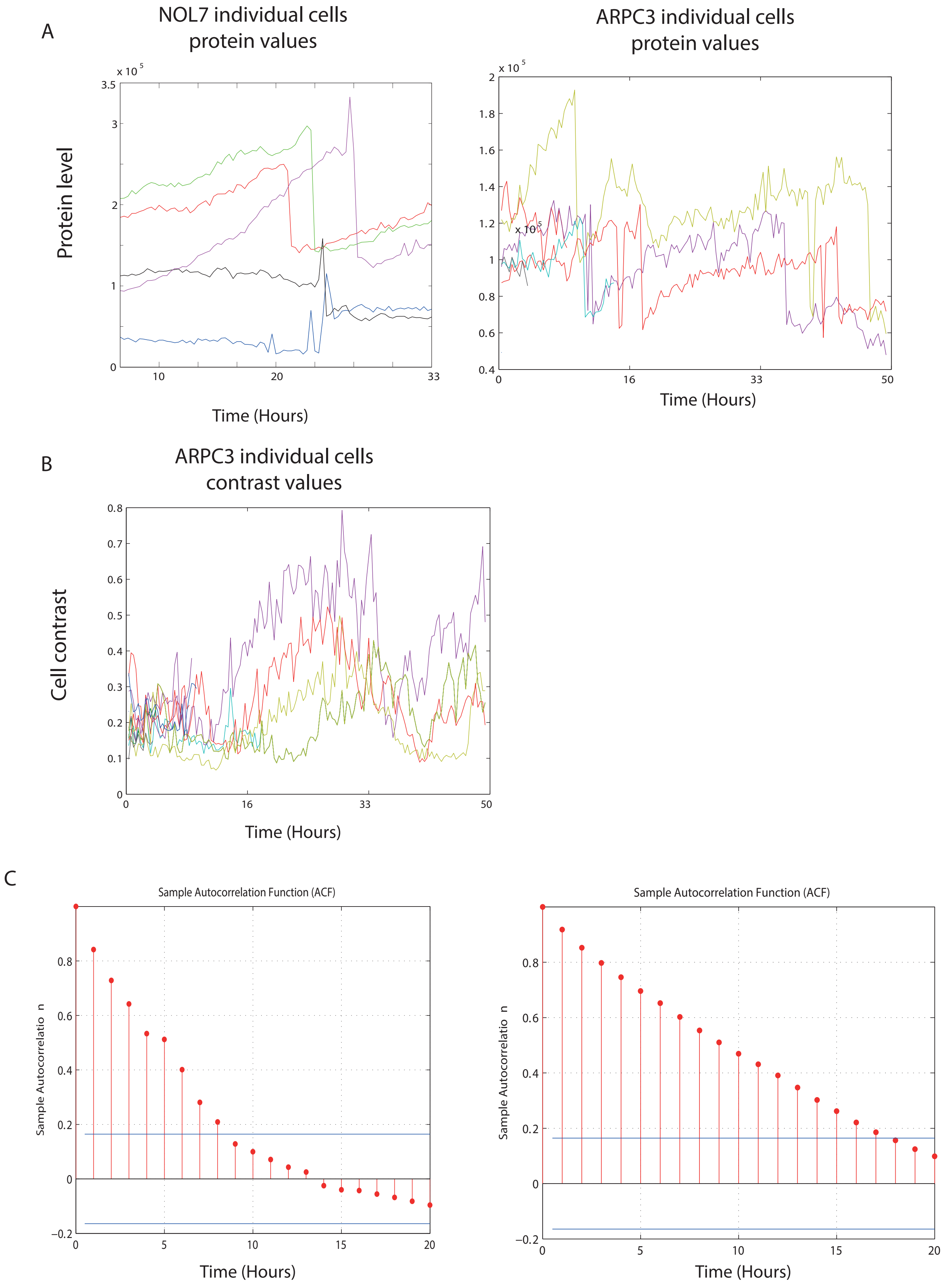
A
NOL7 individual cells
protein values
x 10 5
Protein level
Time (Hours)
ARPC3 individual cells
protein values
x 10 5
Time (Hours)
B
ARPC3 individual cells
contrast values
Cell contrast
Time (Hours)
C
Sample Autocorrelation Function (ACF)
Sample Autocorrelatio n
Time (Hours)
Sample Autocorrelation Function (ACF)
Sample Autocorrelatio n
Time (Hours)
