# Supplementary figures and images for: Noise Genetics: Inferring Protein Function by Correlating Phenotype with Protein Levels and Localization in Individual Human Cells
Source: PLoS Genet. 2014 Mar 6;10(3):e1004176. doi: 10.1371/journal.pgen.1004176 (PMC3945223; doi:10.1371/journal.pgen.1004176)

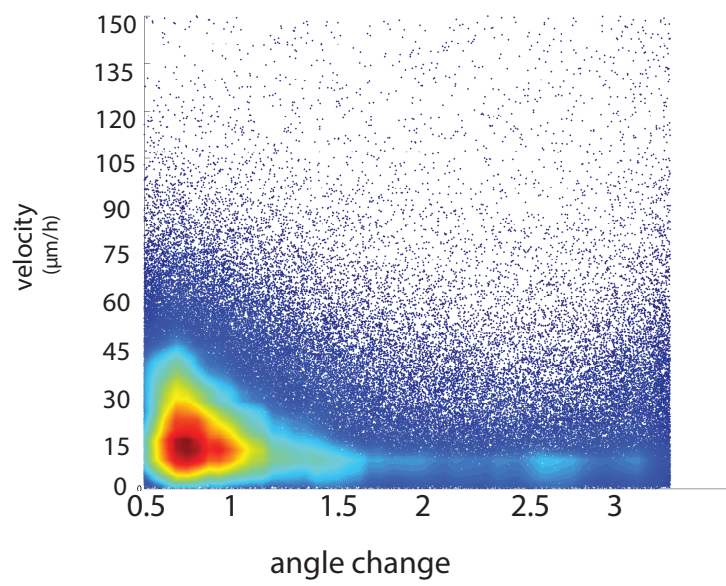

Supplement: Figure S2 — Correlation between velocity and angle change. The velocity and angle change is shown for thousands of cells. Note that a general anti-correlation is evident. (PDF) [file pgen.1004176.s002.pdf]

CALM3

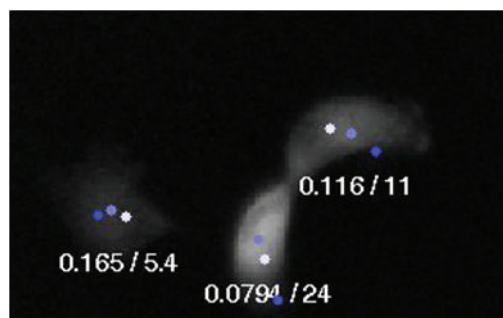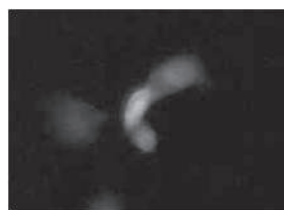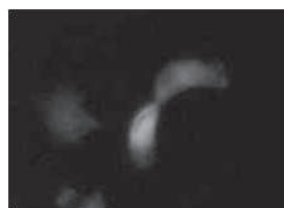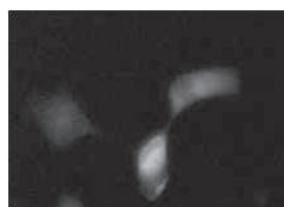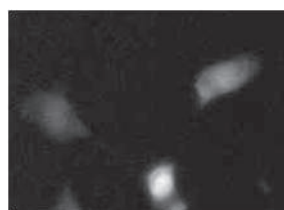

CSNK1A1

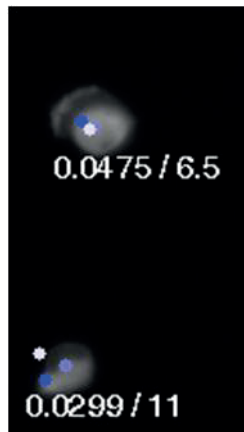

YAP1

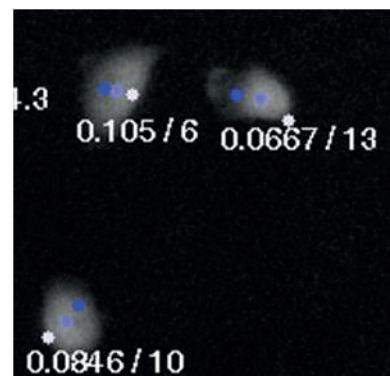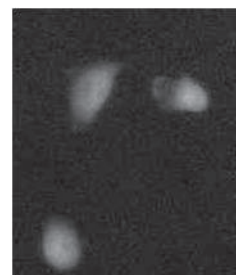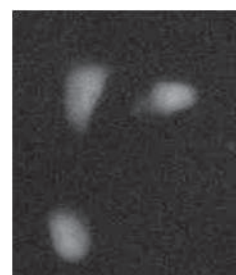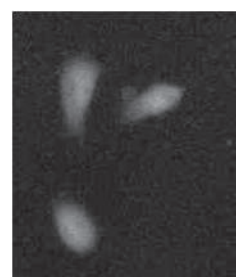

WASF2

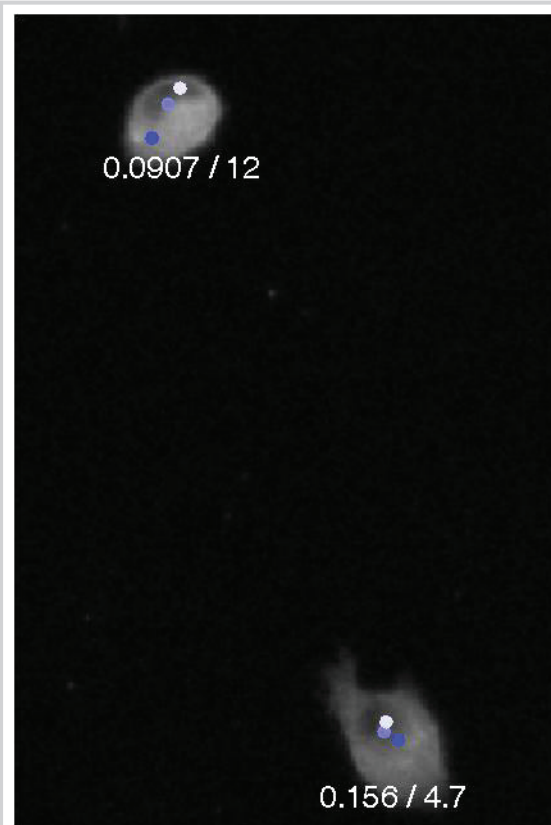

ARPC3

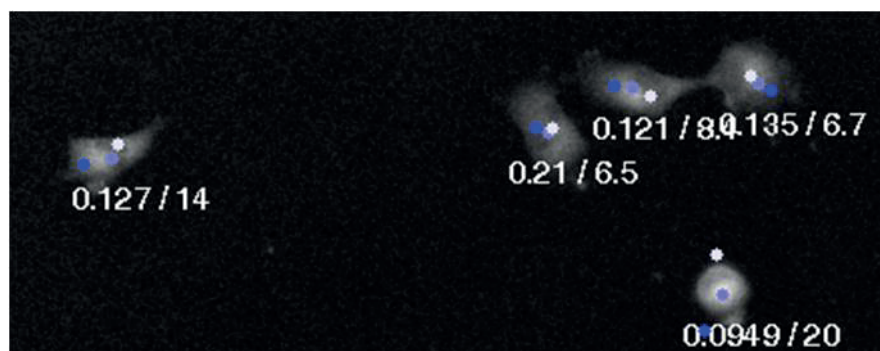

Supplement: Figure S3 — Images of individual cells. Images of individual cells from different clones are shown along with their contrast value (on the left) and their velocity (µM/20 minutes). The white point represents the location of the cell in the previous frame, the light blue point represents the cell location in the current frame and the blue point represents the location in the next frame. (PDF) [file pgen.1004176.s003.pdf]

Cell 2 has lower contrast values when moving faster

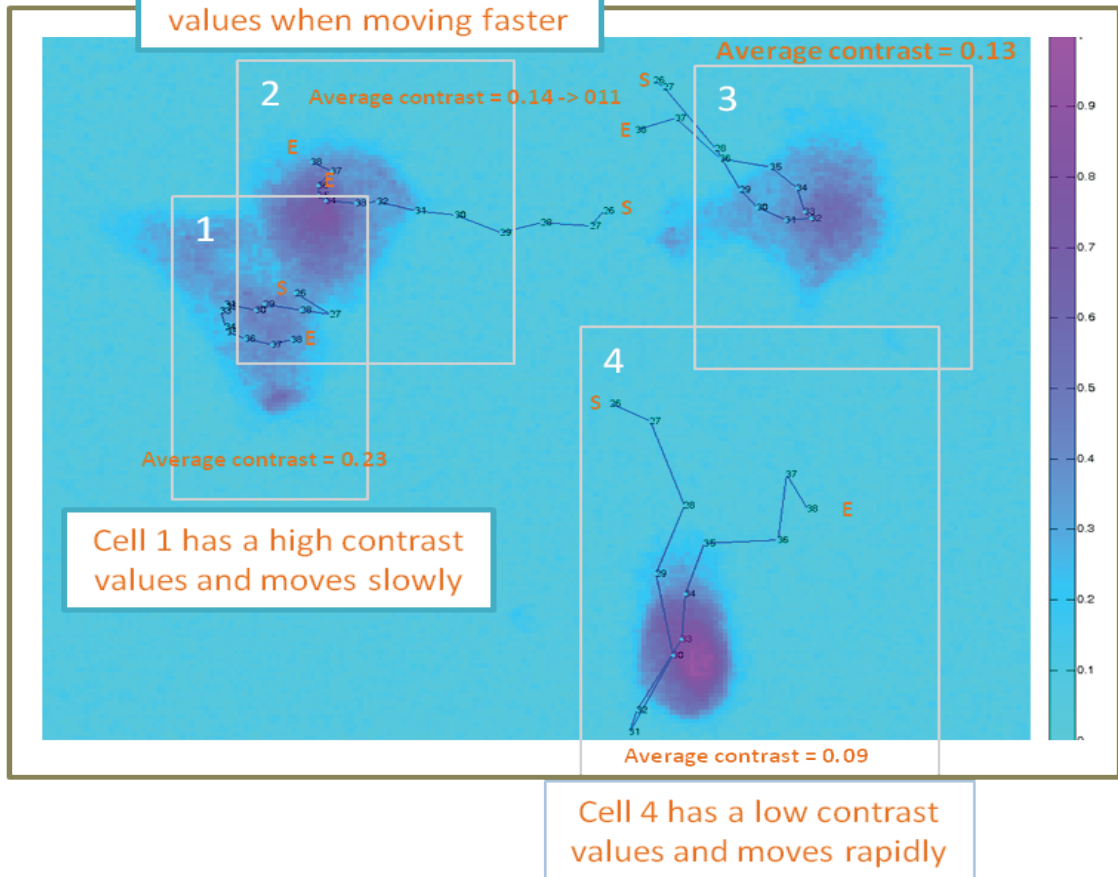

Supplement: Figure S4 — Different individual cells of the ARPC3 clone in one field of view. Four different cells from the ARPC3 clone are shown along with information about their contrast values and their different trajectory along 13 consecutive frames (frames taken every 20 minutes). (PDF) [file pgen.1004176.s004.pdf]

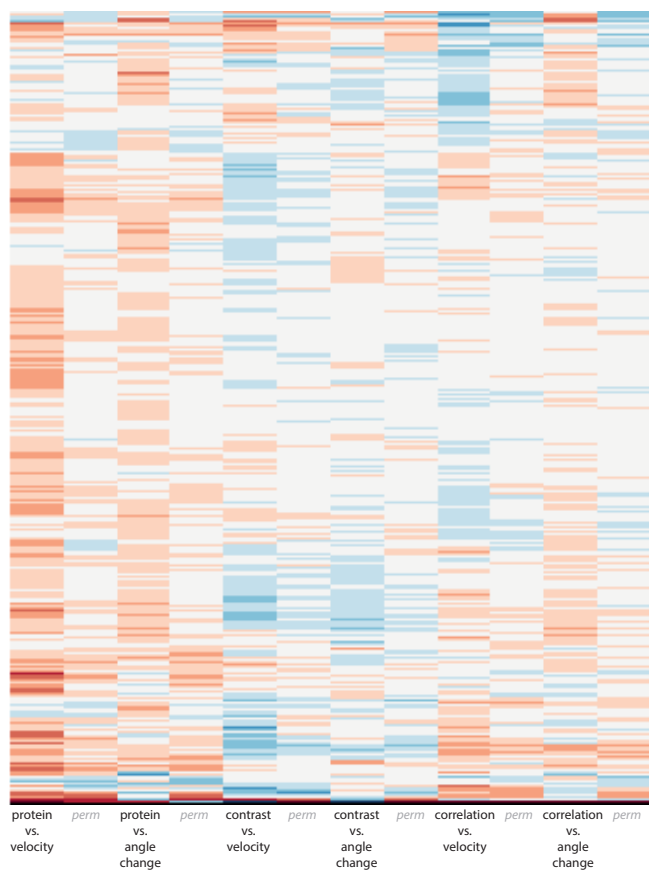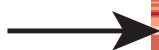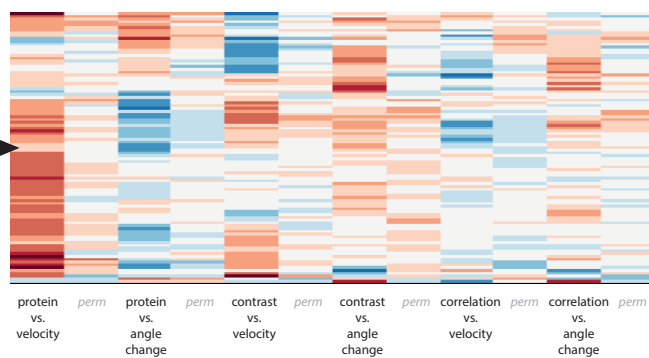

Supplement: Figure S6 — Correlation coefficients R between protein and motility features. Similar to Figure C2, a correlation coefficients matrix between the 3 protein parameters and the two motility features is shown. On the right, a group of proteins with high absolute correlation is shown. Blue/red denotes low/high R values. (PDF) [file pgen.1004176.s006.pdf]

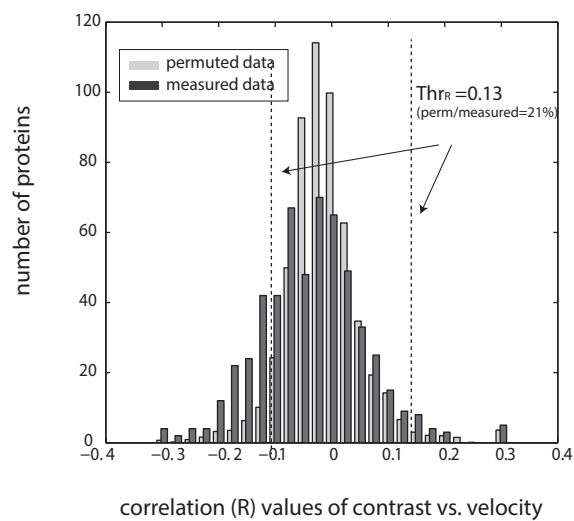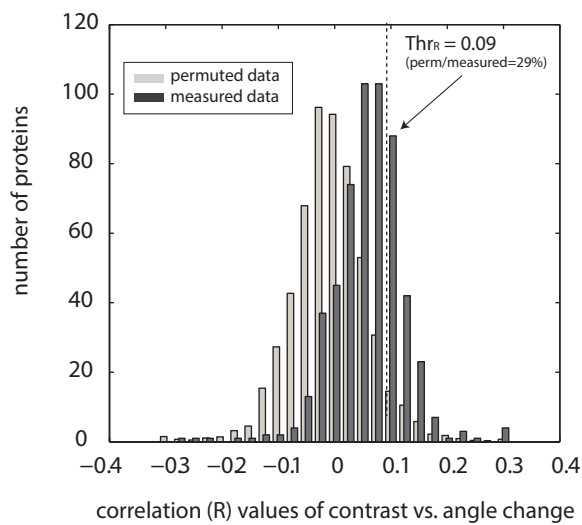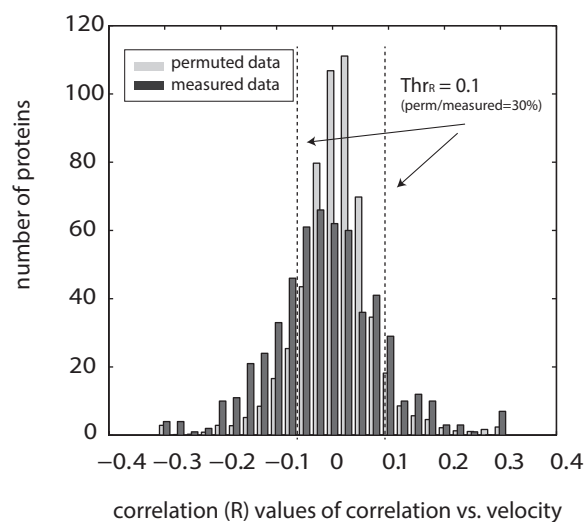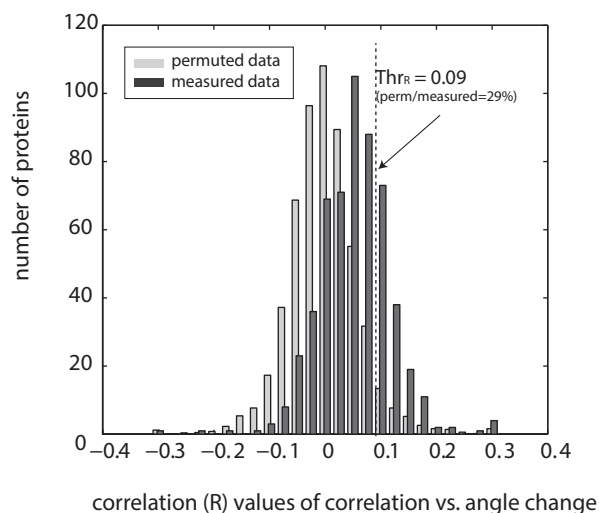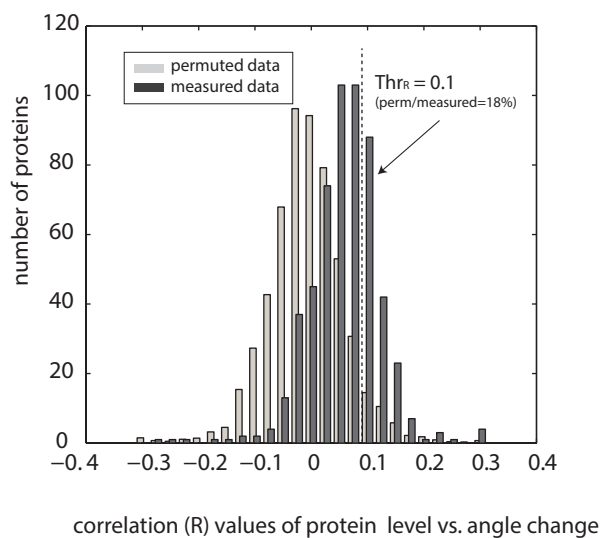

Supplement: Figure S7 — Comparisons between the real and permuted correlation values. The correlation values calculated from the real dataset are in dark grey, while the correlation values calculated from 10 permuted datasets are in light grey. This analysis helped us to choose a threshold that would minimize the number of hits in the permuted dataset compared to the number of hits in the real dataset. The chosen threshold for each comparison is written on the right of the plot. (PDF) [file pgen.1004176.s007.pdf]

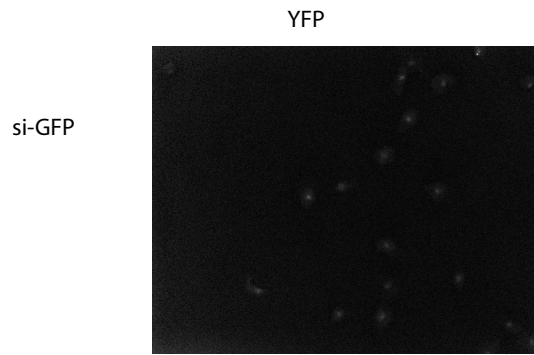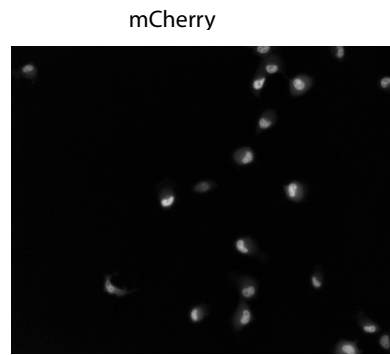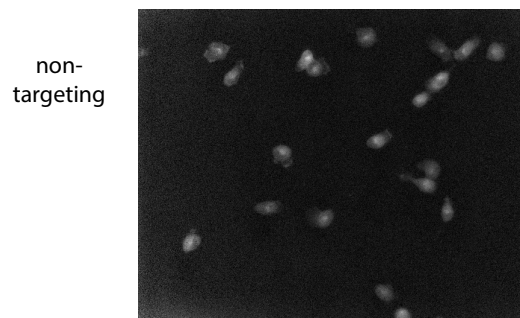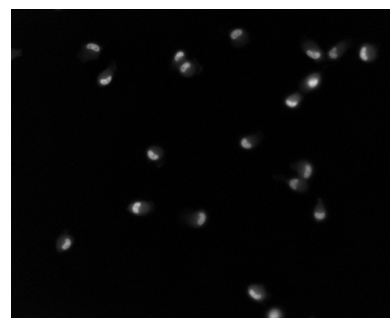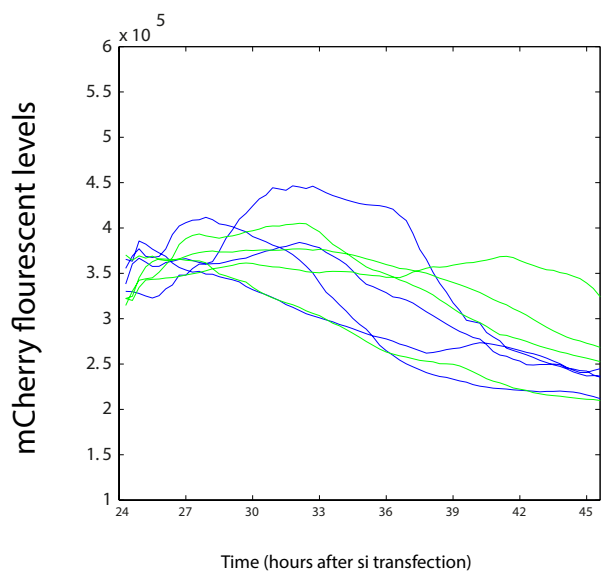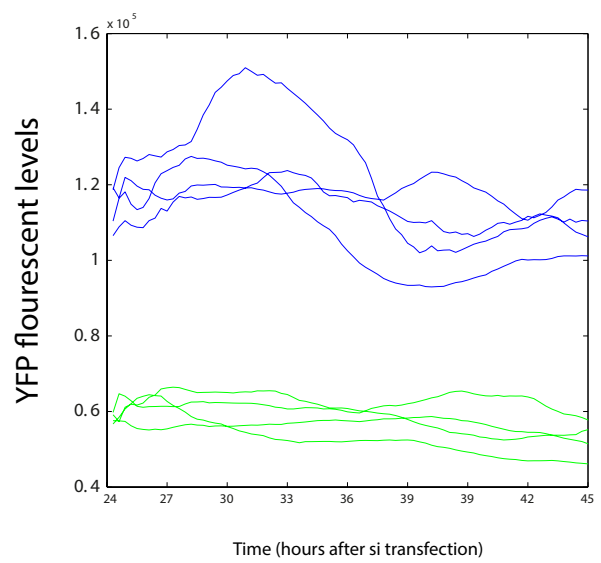

Supplement: Figure S8 — Knockdown experiments specifically decrease the expression of the YFP tagged protein and not the mCherry tagged protein. A typical field of view of the ARPC3 clone is shown after si-GFP experiment and after the control experiment (with non-targeting si). The parental clone has 2 proteins tagged with mCherry to help with the segmentation of the nucleus and the cytoplasm. No decrease in mCherry expression is evident. However, the ARPC3 is tagged with YFP and a significant reduction is shown in the YFP expression. On the bottom, a quantification of 4 different FOVs of the ARPC3 clone demonstrates similar results. Similar results were obtained for all the examined clones. (PDF) [file pgen.1004176.s008.pdf]

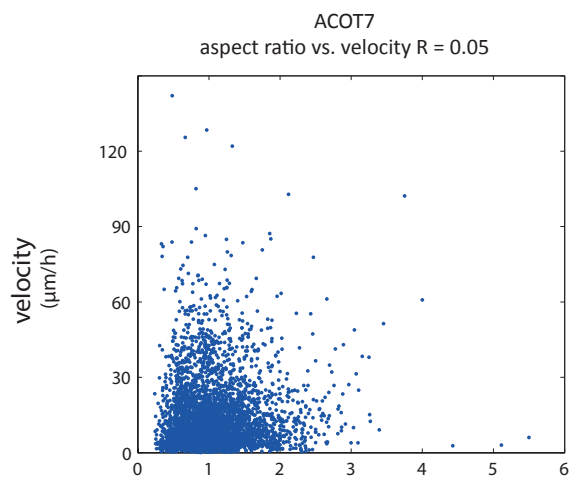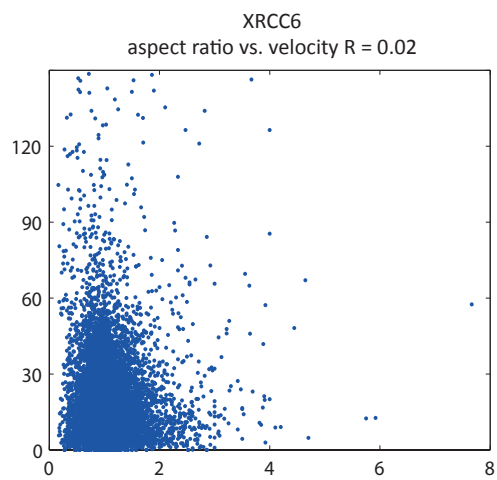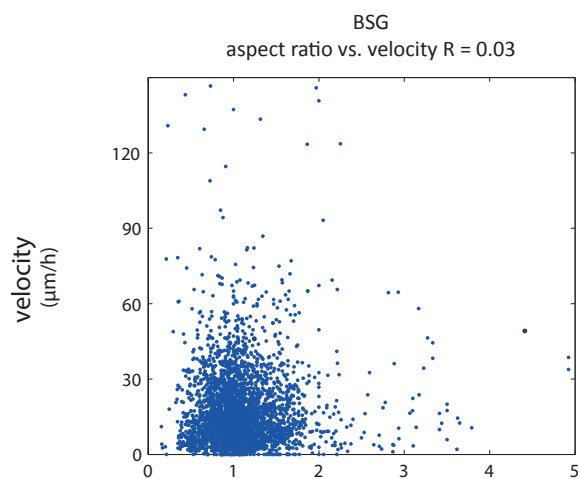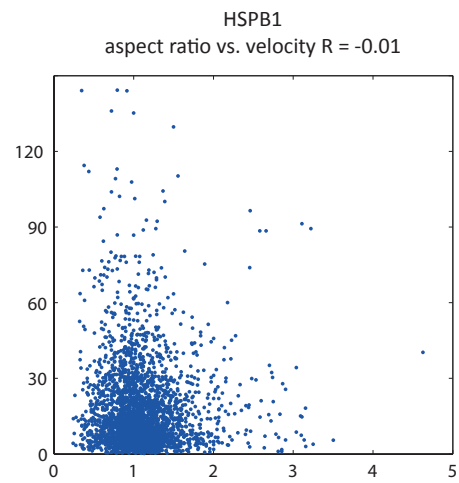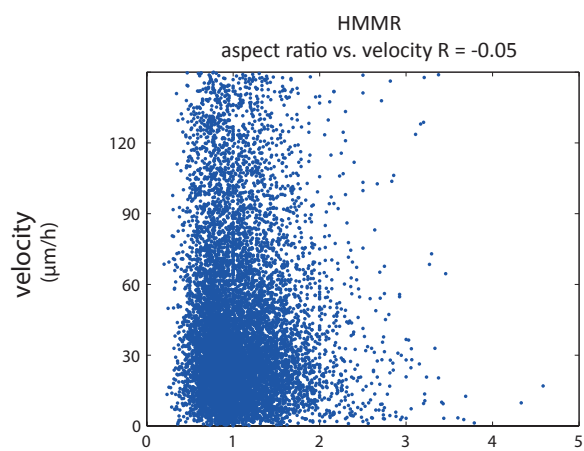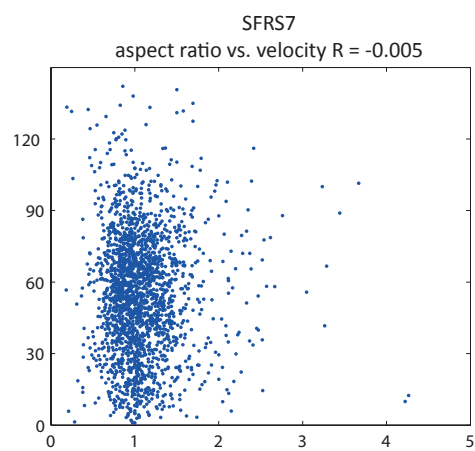

Supplement: Figure S9 — Aspect ratio does not correlate with velocity in our system. The aspect ratio of single cells, a measure that describes cell shape, was plotted against the velocity of cells in the same clones as in figure 2F, E. No significant correlation is evident for any of these clones. (PDF) [file pgen.1004176.s009.pdf]

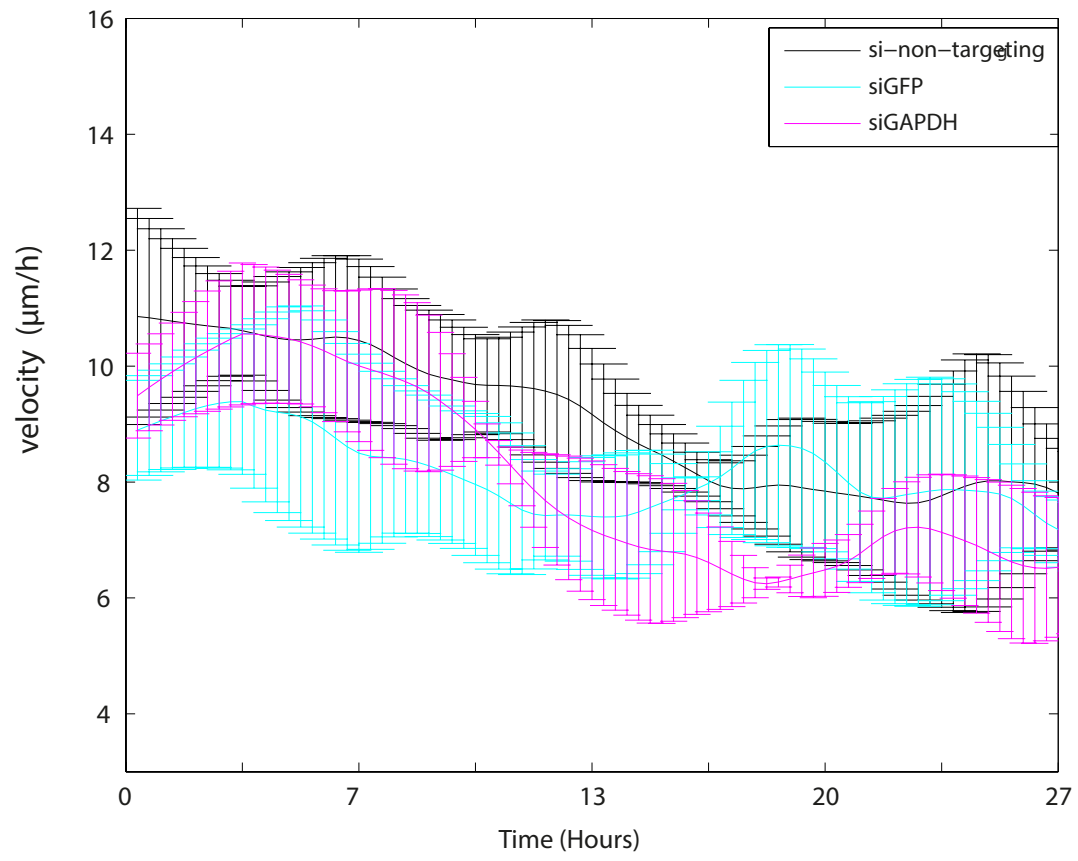

Supplement: Figure S10 — Control knockdown experiments. GAPDH clone was used as a negative control since it is not a candidate motility gene. siRNA against GAPDH (Dharmacon) was used, as well as siRNA against GFP (QIAGEN) that should target any gene in our library that is tagged with YFP and also non-targeting siRNA (Dharmacon) that is not targeted against any specific gene and is widely used as a negative control. As expected, no significant change in the velocity was observed between these 3 conditions. Therefore, in all our following experiment, we used the siGFP that is expected to lower the expression of the target gene and the non-targeting siRNA that serves as a negative control for the siRNA experiment. (PDF) [file pgen.1004176.s010.pdf]

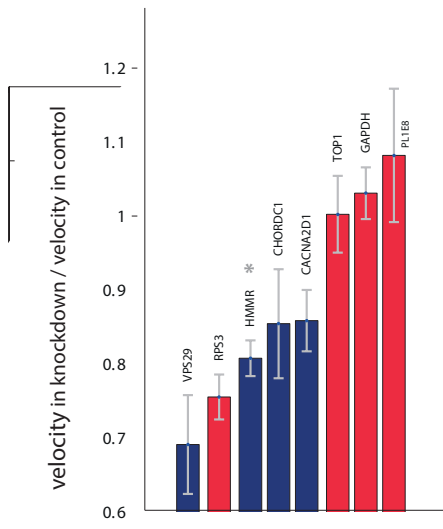

Supplement: Figure S11 — Negative correlation between protein and motility. Knockdown experiments were carried out for 4 genes that showed a negative correlation between protein level and motility. Analysis was done as described in Figure 4C. Velocity reduction in knockdown experiments compared to mock treatment shows that 4/4 candidate genes showed a motility defect upon knockdown (blue bars), in contrast to control genes not known to be involved in motility, for which 3 out of 4 showed no significant defect (red bars). Stars denote known motility genes also found in our assay. Error bars stands for standard deviations (SD). (PDF) [file pgen.1004176.s011.pdf]
