# Supplementary material for: Noise Genetics: Inferring Protein Function by Correlating Phenotype with Protein Levels and Localization in Individual Human Cells
Source: PLoS Genet. 2014 Mar 6;10(3):e1004176. doi: 10.1371/journal.pgen.1004176 (PMC3945223; doi:10.1371/journal.pgen.1004176)
Supplement: Figure S5 — Texture features calculations of images. (A) Cell images from the ARPC3 clone are shown along with their contrast and texture correlation values, calculated without rotation and after averaging the contrast and texture correlation values (after 4 rotations). The contrast is plotted against the velocity for the ARPC3 clone when calculating the contrast without rotation (C) and after averaging the contrast values after 4 rotations (D) A high correlation (R = 0.99) is evident between the contrast values without rotation and the average contrast values after rotation. (E) A high correlation (R = 0.97) is evident between the texture correlation values without rotation and the average texture correlation values after rotation. Similar results were obtained for the different clones. (PDF) [file pgen.1004176.s005.pdf]

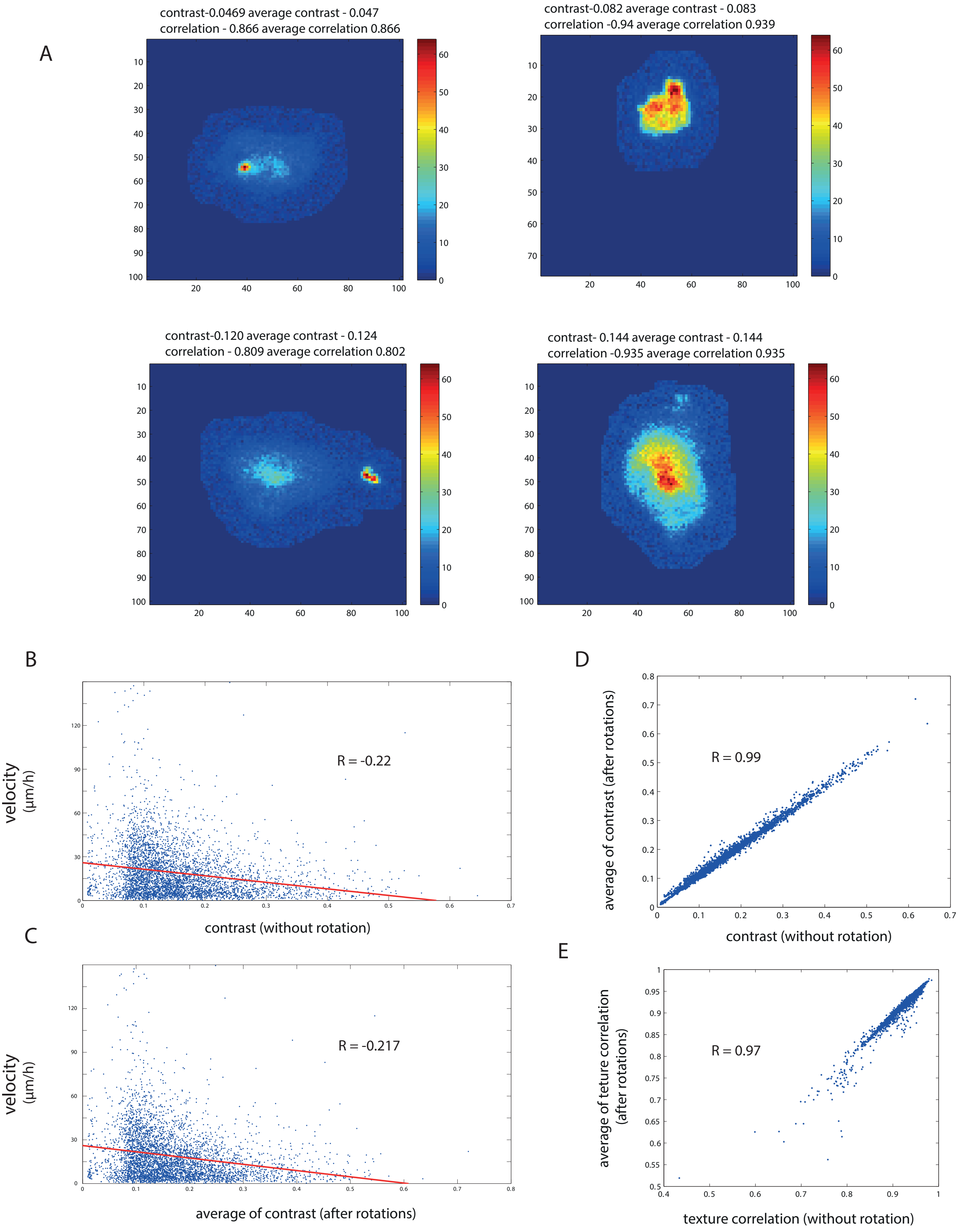
A
contrast-0.0469 average contrast - 0.047
correlation - 0.866 average correlation 0.866
contrast-0.082 average contrast - 0.083
correlation -0.94 average correlation 0.939
contrast-0.120 average contrast - 0.124
correlation - 0.809 average correlation 0.802
contrast- 0.144 average contrast - 0.144
correlation -0.935 average correlation 0.935
B
velocity
(µm/h)
R = -0.22
contrast (without rotation)
C
velocity
(µm/h)
R = -0.217
average of contrast (after rotations)
D
average of contrast (after rotations)
R = 0.99
contrast (without rotation)
E
average of teture correlation
(after rotations)
R = 0.97
texture correlation (without rotation)
