# Supplementary material for: Noise Genetics: Inferring Protein Function by Correlating Phenotype with Protein Levels and Localization in Individual Human Cells
Source: PLoS Genet. 2014 Mar 6;10(3):e1004176. doi: 10.1371/journal.pgen.1004176 (PMC3945223; doi:10.1371/journal.pgen.1004176)
Supplement: File S4 — Negative correlation between protein and motility. A short discussion on genes in our analysis that presented a negative correlation with motility. (DOCX) [file pgen.1004176.s015.docx]

**Negative correlation between protein and motility**

Most of the candidate motility genes in our assay showed a positive correlation between protein level and cell speed. However, about 7% of the candidate proteins (5/74) showed a negative correlation between protein level and cell speed. We thus tested three such negative-correlation genes using mild knockdown (Figure 4C, HMMR, VPS29, CHORDC1). Upon knockdown, we find that their cell speed decreased (instead of increased as expected by the correlation sign) (Figure S11).

One explanation of these findings is that these proteins have a non-monotonic (inverse-u) shaped dependence of motility on protein levels. This U-shaped function has a negative slope near the wild-type protein level and a positive slope at lower levels reached by knockdown experiments. Such a non-monotonic dependence predicts that both removing the gene and over-expressing it can reduce motility. One mechanism for such non-monotonic functions is the incoherent feed-forward loop network motif (Kaplan *et al*, 2008). ]
